# On the Therapeutical Employment of Collodion in Erysipelas, Articular Rheumatism, Puerperal Peritonitis, Etc.

**Published:** 1852-01

**Authors:** M. Robert Latour


### THE NORTH-WESTERN
### MEDICAL AND SURGICAL JOURNAL.

VOL. IV.] JANUARY, 1852. [No. 5.

### Part 1.—Original Communications.

ARTICLE I.

### *On the Therapeutical employment of Collodion in Erysipelas, Articular Rheumatism, Puerperal Peritonitis, etc.:* By M. ROBERT LATOUR.

[We are gratified to see by the following, from the *Revue Medicale Chirurg. of Malgaigne*, which has been translated from the French, for our pages, by Mr. I. P. Lynn, Medical Student, that one at least of our transatlantic cotemporaries is disposed to award the credit of priority in projecting an important improvement, to him to whom honor is due, even though it should be to an American. This is very unlike some persons on both sides of the British Channel, and still others in America who have been disposed to claim the treatment referred to below as of their own projection, although not tried by them for months subsequent to its publication by Dr. Freer, in this Journal, after he had fully and thoroughly tested its virtues.—ED.]

An American Journal announced some months ago, that Dr. J. W. Freer, of Illinois, had used collodion with the happiest results in epidemic erysipelas which prevailed during the last spring. The liquor was spread, by means of a camels-hair pencil, on all the inflamed parts, and was followed by immediate relief and a disappearance of the redness. Dr. Freer has made use of this agent, in the same manner in cases of burns and superficial inflammations, and in every instance he had remarked that the pain was alleviated, whether it acted simply by protecting the inflamed tissue from the action of the air, or, resolution was promoted under the influence of the compression exercised by the collodion.

Since the publication of these facts many French practitioners have repeated the experiments of Dr. Freer and have obtained equally favorable results. M. M. Nelaton, Aran and Lamire have been highly gratified with the effects of this local application. M. M. Aran and Valleix have employed it in variola and they have come to know that, in this affection, the collodion prevents, not only the development of the pustules when applied at the commencement of the disease, but that it arrests them in their evolution even when not applied until the second or third day of the eruptive stage.

But M. Robert Latour has gone much farther with this remedy ; in a paper recently addressed to the Academy of Medicine he exposes the following results:

"Starting from logical reasoning, and not from the chances of empiricism, the employment of the impermeable plaster, directed with so great success against erysipelas and other inflammations of the surface of the body, ought not to remain confined to a circle of diseases thus limited. Confident in the value of the principle which had suggested to me such a therapeutic innovation, I have promptly extended its application to other well marked inflammations; and some months since I informed the Academy of the remarkable facility with which I have subdued by this medication inflammatory action, whether of gout or of acute articular rheumatism. Its success since that time has not diminished; many cases of articular rheumatism and of gout have presented themselves in my practice; and I say it with a just sentiment of pride, that no agent has obtained, by its therapeutic results, a more brilliant sanction A lapse of time of three days at the most, ordinarily of a single day, has invariably sufficed to subdue the disease; and what will secure one against fear of the consequences of so rapid a triumph is the constantly simultaneous disappearance of the general symptoms of the affection. Whatever idea we may have as to the cause of these results, it is necessary to humble ourselves before the fact, *that in no instance, upon being removed, has this local inflammation left behind it any of the general phenomena of the disease.* Certainly this is enough to establish the perfectly innocuous character of such a practice, which should place it in the first rank of therapeutical agents in the affections which I now come to describe."

We attach a little less importance than the author, to this *logical reasoning*, and, therefore, we pass over some of these reasonings. We shall find sufficient of them in the recital of facts which follow. The first of which is a case of puerperal peritonitis:

"Miss X, a strong, healthy young woman of 22 years, after having been exposed to cold for a considerable length of time, was seized with a very severe chill which lasted an hour and to which succeeded a burning fever, great anxiety, cephalalgy and pain in the abdomen. The pain, increasing and extending itself on the next day had become general and so acute that she could not endure the slightest touch. The abdomen was tumefied, the respiration humid, fever high, with extreme restlessness, and to all these symptoms was added vomiting which became more and more frequent. The development of a peritonitis here could not be doubted, and the indication was plain, according to orthodox rules, to proceed to the extraction of large quantities of blood. Such a step would certainly have been legitimate, and has been so often proved as to place it above all imputation. But, true to the theory which I have developed touching the philosophy of inflammation, I conceived the idea of removing the disease by animal heat, and without drawing a drop of blood, I coated the entire abdomen with collodion. Here, I confess, I experience some embarrassment in stating the results obtained. To say that the vomiting was immediately arrested, that in two hour's time calmness had taken the place of extreme anxiety, that the pain was alleviated ; to say that the skin in less than a day had recovered its natural moisture and coolness, and the pulse its normal condition, from having been at 112 ; to say, in short, that less than twenty-four hours were sufficient to subdue this alarming inflammation, is sufficient to destroy any incredulity that may exist on the part of the schools in regard to my philosophy of the disease and the indications of treatment."

This is not the only time that the author has employed this agent in this affection, and in every case, he says it has produced the happiest effects. It is well known, however, that if the disease is already complicated with effusions and adherences it will not immediately remove these disorders ; but even then M. Latour affirms that it arrests the progress of the disease.

"The following will show the advantage which I derived in the case of a lady, aged forty-five years, of enfeebled health, and who, while laboring under a chronic ovaritis of the right side, was attacked with a general peritonitis. The serious nature of which was evident by extreme pain throughout the abdomen, violent fever, anxiety and vomiting, which, having commenced on the third day after the attack, on the next day became excessively frequent, at which time my services were solicited. At this period the abdomen was greatly tumified in spite of the great quantity of blood extracted, by the application of twenty leeches, the night previous; and the patient, in extreme agitation, most imploringly demanded relief. She obtained it. The bleeding from the leech-bites was immediately stopped and collodion applied over the whole surface of the abdomen. This application sufficed, in a few hours, to arrest the vomiting and moderate the pain, and if convalescence was not evidently established the same day the *life*, at least, *was saved*, which, but a short time before, was so threatened and already even compromised. Some days were necessary to restore her to her former condition, which was but a state of disease and suffering, occasioned by the chronic ovaritis, and which was removed only by long continued treatment."

We wait, for the present, the exposition of facts. They should be repeated and submitted to rigorous verification; this first being done, it will then be time to examine and appreciate the theory which has furnished the author a point of departure.

